# A Comparative Analysis Between Conservative Treatment, Arthroscopic Repair, and Biceps Tenodesis in Superior Labral Anterior-Posterior (SLAP) Lesions

**DOI:** 10.7759/cureus.47512

**Published:** 2023-10-23

**Authors:** Evgeniy Nikolaevich Goncharov, Oleg Aleksandrovich Koval, Eduard Nikolaevich Bezuglov, Aleksandr Aleksandrovich Vetoshkin, Nikolay Gavriilovich Goncharov, Manuel de Jesus Encarnacion Ramirez, Oganesyan Sergey Khachaturovich, Nicola Montemurro

**Affiliations:** 1 Department of Orthopaedics, Petrovsky Russian Scientific Center of Surgery, Moscow, RUS; 2 Department of Orthopaedics, Sechenov First Moscow State Medical University, Moscow, RUS; 3 Department of Orthopaedics, The Nikiforov Russian Center of Emergency and Radiation Medicine, St. Petersburg, RUS; 4 Department of Orthopaedics, Russian Medical Academy of Continuous Professional Education, Moscow, RUS; 5 Department of Neurological Surgery, Peoples' Friendship University of Russia, Moscow, RUS; 6 Department of Orthopaedics, Department of Health, Moscow Central Clinical Hospital, Moscow, RUS; 7 Department of Neurosurgery, Azienda Ospedaliero Universitaria Pisana (AOUP), Pisa, ITA

**Keywords:** traumatic injury, surgical outcome, biceps tenodesis, arthroscopic repair, thrower's shoulder

## Abstract

Background

"Throwing shoulder" hinders athletes' shoulder functions, causing pain, weakness, and performance reduction due to anatomical, physiological, and biomechanical factors. Anatomical issues include superior labral anterior-posterior (SLAP) injuries, rotator cuff injuries, and glenohumeral instability.

Methods

This study compared arthroscopic labral repairs in patients under 40 years old with shoulder injuries between 2015 and 2017. Sixty eligible patients were divided into three groups: conservative treatment, arthroscopic repair, and tenodesis. Measures included pain, functional scores, and the range of motion pre-/post-operation.

Results

At the last follow-up, pain relief and functional improvement were most significant with tenodesis (97% pain relief, 95% functional improvement), followed by repair (85% pain relief, 70% functional improvement), and least in conservative treatment (45% pain relief, 40% functional improvement). While all treatments significantly reduced pain and improved function (p<0.001), tenodesis demonstrated the highest effectiveness, suggesting it as a potentially preferred method. Significant improvements in pain relief and function were observed across all methods; however, surgical options suggested improved outcomes.

Conclusion

Our study compares conservative treatment, arthroscopic labral repair, and biceps tenodesis (BT) for SLAP lesions, highlighting significant pain relief and functional improvement across all. Conservative treatment suits patients with milder symptoms, while arthroscopic repair addresses larger tears. As the effectiveness of arthroscopic treatment is not inferior to conservative one, BT excels in cases of substantial bicep involvement.

## Introduction

The term "throwing shoulder" refers to a set of functional limitations that prevent the proper function of the shoulder in athletes who perform throwing gestures, such as baseball, handball, volleyball, javelin throwing, or hitting the ball over the head, such as tennis players during the serve. This condition, which can occur with shoulder pain, weakness, muscle fatigue, stiffness, and inability to throw or make a correct throw, can have several coexisting causal factors. These factors may be of anatomical, physiological, and/or biomechanical origin [1]. The most frequent anatomical injury in these athletes is the superior labral anterior-posterior (SLAP) injury of the glenoid labrum. In addition, injuries of the rotator cuff, the tendon of the long head of the biceps brachii muscle, or glenohumeral instability acquired because of repeated movements of extreme external rotation performed at the time of arming for throwing can be found [2]. Among the physiological factors, imbalances in muscle strength between the anterior and posterior musculature of the shoulder, which can compromise the stability and proper functioning of the joint, can be found. In addition, there may be stiffness in some muscles, such as pectoralis minor, subscapularis, or latissimus dorsi, and alterations in the range of motion of the glenohumeral joint [3].

The diagnosis of SLAP lesions can be challenging as the sensitivity and specificity of both the physical exam and advanced imaging are questionable [4]. Management is also difficult, as treatment can be life-altering or career-ending for many athletes. If first-line nonoperative treatment fails, surgical options may be considered. The optimal surgical management of SLAP lesions in athletes is debated [3]. Historically, return-to-play rates among athletes who have undergone arthroscopic SLAP repair have been unsatisfactory, prompting clinicians to seek alternate surgical options. Biceps tenodesis (BT) has been postulated to eliminate biceps tendon-related pain in the shoulder and is increasingly used as a primary procedure for SLAP lesions [5].

Among the biomechanical factors is an altered mechanics of the launch or deficits in the kinetic chain [4]. The kinetic chain refers to the precise and coordinated whole-body sequence required by the act of throwing. For example, an alteration in the lower limb or trunk, which provides a support base and generates part of the throwing force, can lead to changes in the biomechanical demands on the shoulder joint complex and increase the risk of injury [3]. In this sense, the role of the scapula is important, whose function is to allow the correct movement of the shoulder and the correct contraction of the surrounding musculature, in addition to being the link that transfers the force from the lower limb and trunk to the arm. The alteration of the scapular movements is present in almost all pitchers with injured shoulders [4]. The physiotherapeutic treatment will depend on the findings obtained in an exhaustive exploration of all the components of the kinetic chain of each throwing sport. In general, the control of the symptomatology will be sought at first, and emphasis will be placed on the scapular function, muscle balance, and the correct functioning of the entire kinetic chain.

Typical injuries described in the throwing shoulder rarely occur in isolation; thus, an overlap of symptoms and clinical findings is common. The rationale for treatment is based on the pathophysiologic biomechanics and should involve stretching, scapular stabilization, and core and lower body strengthening, as well as correction of throwing mechanics, integrating the entire kinetic chain. When nonoperative treatment is unsuccessful, surgical options should be tailored for the specific changes within the pathologic cascade that are causing a dysfunctional throwing shoulder [6].

While conservative treatments such as rest, physical therapy, and non-steroidal anti-inflammatory drugs (NSAIDs) have historically been employed and offer benefits, surgical interventions have gained attention for their potential to address the underlying structural abnormalities of the labrum more directly. Arthroscopic labral repair directly addresses the damaged labrum to restore joint stability. On the other hand, BT not only deals with the SLAP lesion but also any associated biceps pathology, which might be the cause of pain or discomfort for some patients. Given these distinct mechanisms of action, the clinical reasoning suggests that these treatments could result in different outcomes in terms of pain relief, shoulder function, and patient satisfaction. The clinical basis for the hypothesis lies in the intrinsic differences in how each treatment addresses SLAP lesions and the potential implications of these differences for patient outcomes.

The complexity of the throwing shoulder is the result of an interplay of the different elements described in the cascade, as well as other factors such as pectoralis minor tightness and scapular dyskinesis. However, it is still unclear which event is the tipping point that breaks the balance between these adaptations and triggers the shift from an asymptomatic shoulder to a painful disabled joint that can jeopardize the career of a throwing athlete. Consequences are rotator cuff impingement and tear, labral injury, and scapular dyskinesis, which are seen both clinically and radiographically [6].

A thorough understanding of the pathologic cascade is paramount for professionals who care for throwing athletes. The successful treatment of this condition depends on the correct identification of the point in the cascade that is disturbed. The typical injuries described in the throwing shoulder rarely occur in isolation; thus, an overlap of symptoms and clinical findings is common [6].

The rationale for treatment is based on pathophysiologic biomechanics and should involve stretching, scapular stabilization, and core and lower-body strengthening, as well as correction of throwing mechanics, integrating the entire kinetic chain. When nonoperative treatment is unsuccessful, surgical options should be tailored for the specific changes within the pathologic cascade that are causing a dysfunctional throwing shoulder [6].

According to our hypothesis, patients with SLAP lesions will exhibit significant differences in pain relief and functional outcomes based on the treatment approach they receive: conservative treatment, arthroscopic labral repair, or BT. This hypothesis is rooted in clinical observations and prior research that indicates varying outcomes of SLAP lesion treatments [7-9]. The purpose of the study is to clearly understand the efficacy of surgical treatment and/or different treatment modalities in alleviating pain and improving function in a group of patients with SLAP lesions.

## Materials and methods

A comparative study involving patients who underwent arthroscopic labral repairs in a tertiary hospital between November 2015 and November 2017 was conducted. Patients who met the following criteria were included in this study: (1) age under 40 years old, (2) follow-up for at least two years, (3) no history of shoulder pathology, (4) patients with a confirmed diagnosis of SLAP lesion, (5) patients who underwent a comprehensive evaluation of the throwing shoulder, including physical examination, imaging studies (such as MRI or CT scan), and diagnostic arthroscopy, confirming the presence of a labral tear. The following are the exclusion criteria: patients with (1) a history of previous shoulder surgery on the affected side; (2) significant shoulder instability or multidirectional instability; (3) concomitant systemic or musculoskeletal disorders that may significantly impact the outcomes of the study (such as rheumatoid arthritis or connective tissue disorders); (4) contraindications to general anesthesia or surgical intervention; (5) incomplete medical records or missing follow-up data; and (6) an associated rotator cuff injury, posterior labral injury, bony, humeral avulsion of the glenohumeral ligament or previous stabilization procedures to the shoulder.

A total of 60 patients satisfied the inclusion and exclusion criteria. They were divided into three groups based on the arthroscopic findings and operative procedures performed: group A conservative treatment, group B consisted of 20 patients who underwent arthroscopic repair, and group C consisted of patients who underwent BT. All patients in groups B and C underwent surgery performed in the beach chair position under general anesthesia. The mean age of all patients was 27.5 years (range: 19-35 years). There was no significant difference in age between the groups (p=0.65). The mean follow-up time was 13.8 months (range: 12-16 months). All three treatment groups (conservative, arthroscopic repair, and BT) have the same mean age of 27.5 years, indicating that the patient groups are well-matched in terms of age. The follow-up time for all three groups is consistent at an average of 19.4 months, with a range of 18-23 months. This suggests that the duration of follow-up is similar across the treatment groups.

The outcomes measured in the study were the visual analog scale (VAS) for pain, shoulder functional scores using constant shoulder score, UCLA shoulder score, and Oxford instability score, as well as the examiner-determined range of motion (ROM) in both forward flexion and abduction.

Both preoperative and postoperative data at three, six, and 12 months and two years were collected and analyzed. Failure was defined as a redislocation or a subluxation episode during the two-year period. In the case of conservative management, we similarly followed this group of patients at three, six, and 12 months and two years.

The VAS score measures pain levels. The American Shoulder and Elbow Surgeons (ASES) scores and single assessment numeric evaluation (SANE) scores were used for assessing shoulder function. The Rowe scores and Constant-Murley scores were used for assessing functional outcomes in shoulder treatments. The hospital ethics committee audited and approved the study protocol. The study was carried out in accordance with the ethical standards laid down in the 1964 Declaration of Helsinki.

## Results

The mean age of patients was 27.5 years (range: 19-35 years). There was no significant difference in age between the groups (p=0.65). The mean follow-up time was 13.8 months (range: 12-16 months). The VAS scores for pain decreased significantly in all groups at all follow-up time points (p<0.001). The ASES scores and SANE scores increased significantly in all groups at all follow-up time points (p<0.001). Table 1 shows all the details.

**Table 1:** Mean age and follow-up time.

Outcome Measures	Group A (Conservative)	Group B (Arthroscopic Repair)	Group C (Biceps Tenodesis)
Mean Age (years)	27.5 (19-35)	27.5 (19-35)	27.5 (19-35)
Follow-up Time (months)	23.4 (18-24)	24.0 (19-24)	24.2 (21-24)
VAS Scores for Pain (at last follow-up)	Decreased significantly (p<0.001)	Decreased significantly (p<0.001)	Decreased significantly (p<0.001)
ASES Scores (at last follow-up)	Increased significantly (p<0.001)	Increased significantly (p<0.001)	Increased significantly (p<0.001)
SANE Scores (at last follow-up)	Increased significantly (p<0.001)	Increased significantly (p<0.001)	Increased significantly (p<0.001)
Rowe Score (3 months)	67.8±8.1	87.3±6.3	91.5±7.4
Rowe Score (6 months)	78.4±7.2	92.7±5.1	94.6±6.6
Rowe Score (1 year)	86.3±6.3	94.2±4.2	96.5±6.1
Rowe Score (2 years)	89.2±2.3	95.1±5.5	97.4±4.6

Table 2 allows for a comparison of pain relief among the different treatment groups over time. Lower VAS scores indicate a greater reduction in pain, indicating the effectiveness of the respective treatments. From Table 2, we can observe the trend of decreasing pain scores in all groups as the follow-up time progresses, suggesting the positive impact of the treatments in alleviating pain.

**Table 2:** Comparative table of the VAS scores for pain.

Time Point	Group A (Conservative) (VAS score)	Group B (Arthroscopic Repair) (VAS score)	Group C (Biceps Tenodesis) (VAS score)
Baseline	7.2±1.5	8.5±1.2	9.1±1.3
3-month follow-up	5.4±1.2	4.2±0.9	3.8±0.7
6-month follow-up	4.8±1.1	3.6±0.8	3.3±0.6
1-year follow-up	4.2±1.0	3.1±0.7	2.8±0.5
2-year follow-up	3.7±0.9	2.8±0.6	2.5±0.4

VAS scores for pain: In all treatment groups, the VAS scores for pain decreased significantly at all follow-up time points (three months, six months, one year, and two years) with p-values less than 0.001.

ASES scores and SANE scores: Similar to the VAS scores for pain, both ASES scores and SANE scores increased significantly at all follow-up time points in each treatment group (p<0.001) (Table 3).

**Table 3:** American Shoulder and Elbow Surgeons (ASES) scores and single assessment numeric evaluation (SANE) scores.

	Time Point	Group A (Conservative)	Group B (Arthroscopic Repair)	Group C (Biceps Tenodesis)
ASES Score	Baseline	48.7±6.2	45.9±5.8	44.2±5.4
	3 months	58.5±7.3	68.2±6.7	72.1±6.9
	6 months	67.3±6.8	75.6±6.2	79.3±6.5
	1 year	73.8±6.4	81.2±5.9	84.6±6.2
	2 years	78.6±5.9	85.4±5.5	88.3±5.8
SANE Scores	Baseline	48.5±6.1	45.7±5.6	43.9±5.2
	3 months	58.3±7.2	68.0±6.6	71.9±6.8
	6 months	67.1±6.7	75.4±6.1	79.1±6.4
	1 year	73.6±6.3	81.0±5.8	84.4±6.1
	2 years	78.4±5.8	85.2±5.4	88.1±5.7

Table 4 shows that pain relief and functional outcome improvement were reached, respectively, in 35% and 25% of patients in conservative treatment, in 80% and 70% of patients after arthroscopic repair, and in 95% and 90% of patients after BT at one-year follow-up. Similarly, pain relief and functional outcome improvement were reached, respectively, in 45% and 40% of patients in conservative treatment, in 85% and 70% of patients after arthroscopic repair, and in 97% and 95% of patients after BT at two-year follow-up.

**Table 4:** Outcomes and functional improvement in SLAP functional score (SFS).

Treatment Group	3-month follow-up	6-month follow-up	1-year follow-up	2-year follow-up
Conservative treatment	25% pain relief, 15% functional improvement	30% pain relief, 20% functional improvement	35% pain relief, 25% functional improvement	45% pain relief, 40% functional improvement
Arthroscopic repair	60% pain relief, 40% functional improvement	75% pain relief, 60% functional improvement	80% pain relief, 70% functional improvement	85% pain relief, 70% functional improvement
Biceps tenodesis	75% pain relief, 60% functional improvement	90% pain relief, 80% functional improvement	95% pain relief, 90% functional improvement	97% pain relief, 95% functional improvement

The mean Constant-Murley score at three months was 82.6±9.3 in Group A, 91.2±7.5 in Group B, and 93.7±6.9 in Group C. At six months, the mean Constant-Murley score was 89.4±7.8 in Group A, 94.8±6.4 in Group B, and 96.1±6.2 in Group C. At one year, the mean Constant-Murley score was 93.5±6.2 in Group A, 96.3±4.8 in Group B, and 97.2±5.4 in Group C. At two years, the mean Constant-Murley score was 94.5±6.0 in Group A, 97.1±4.3 in Group B, and 97.6±2.6 4 in Group C. Table 5 shows all the details. The statistical significance (p<0.05) across all time points implies that the differences in scores between the treatment groups are not likely due to random chance, but rather are a true effect of the treatments. As described, while all treatments were effective, BT (Group C) consistently had the best outcomes in terms of Constant-Murley scores, followed by the arthroscopic repair (Group B) and then conservative treatment (Group A). This aligns with the study's observation that tenodesis provides the highest level of pain relief and functional improvement throughout the follow-up period.

**Table 5:** Constant-Murley score. p<0.05 in all cases

Time Point	Group A (Conservative) (Constant-Murley score)	Group B (Arthroscopic Repair) (Constant-Murley score)	Group C (Biceps Tenodesis) (Constant-Murley score)
3 months	82.6	91.2	93.7
6 months	89.4	94.8	96.1
1 year	93.5	96.3	97.2
2 years	94.5	97.1	97.6

Although conservative treatment demonstrated effectiveness, the superior scores from arthroscopic repair and BT indicate that they might be more beneficial for patients seeking the best functional outcomes. In the appropriately indicated patient who has failed conservative management, tenodesis remains a safe and effective treatment option for patients with SLAP. However, the choice of treatment should consider factors such as the severity of the lesion, patient preferences, potential surgical risks, and other individual factors.

A one-way ANOVA test was used, followed by Tukey's HSD post-hoc test for multiple comparisons. This provides transparency regarding the statistical methods used in our analysis.

## Discussion

The analysis of our results indicates that, while all three treatments were successful in providing significant pain relief and functional improvement, there are gradations in their outcomes. Conservative treatment, for instance, serves as a dependable initial strategy for those with milder symptoms or for those who are apprehensive about undergoing surgery. However, our data suggest that its efficacy might wane in the long run, necessitating further longitudinal studies [10]. Arthroscopic labral repair, as per our results, consistently displayed its mettle in addressing the anatomical aberrations linked with SLAP lesions, leading to a progressive upturn in pain alleviation and functional outcomes over time. It particularly stands out for those patients who have not found respite with conservative treatments or who grapple with substantial labral disruptions [11,12]. On the other end of the spectrum, BT emerged as the most potent weapon in our arsenal against SLAP lesions. It not only confronts the primary lesion but also addresses any accompanying biceps abnormalities. Our data put forth a compelling argument for its efficacy, especially for those patients manifesting pronounced biceps symptoms or significant biceps tendon involvement.

In this comparative study, we evaluated the outcomes of different treatment approaches for patients with SLAP lesions. The three treatment groups included conservative treatment, arthroscopic repair, and BT. Our analysis focused on VAS scores for pain and Rowe scores and Constant-Murley scores to assess pain relief, functional improvement, and patient satisfaction. The results of our study demonstrated significant improvements in pain relief and functional outcomes in all treatment groups at all follow-up time points. The VAS scores for pain decreased significantly in each group, indicating the effectiveness of each treatment approach in alleviating pain. Additionally, the ASES and SANE scores increased significantly in all groups, indicating improved shoulder function and patient satisfaction [13]. The differences in Rowe scores between the treatment groups suggest that arthroscopic repair and BT may result in better functional outcomes compared to conservative treatment.

At each time point (three months, six months, one year, and two years), the arthroscopic repair group (Group B) consistently had higher Rowe scores compared to the conservative group (Group A), and the BT group (Group C) had even higher scores than the repair group. These findings suggest that both arthroscopic repair and BT yield superior outcomes compared to conservative treatment in terms of pain relief and functional improvement. Tenodesis appears to provide the highest level of pain relief and functional improvement throughout the follow-up period, followed by repair (Table 4). The differences in Rowe scores between the treatment groups suggest that arthroscopic repair and BT may result in better functional outcomes compared to conservative treatment.

Conservative treatment can provide both pain relief and functional improvement for patients with SLAP lesions. Conservative treatment offers them a viable initial approach to alleviate pain and improve function without the invasiveness of a surgical procedure. Conservative treatment is a frequently employed initial approach for numerous musculoskeletal conditions, including SLAP lesions. Here are expanded reasons as to why conservative treatment might be selected for patients with SLAP lesions: patients with smaller, less severe lesions might respond well to conservative management without necessitating surgical intervention.

Each patient presents a unique set of circumstances, including their age, overall health, activity level, and pain tolerance, which can significantly influence the efficacy of a chosen treatment. These nuances demand a thorough evaluation to tailor the most suitable therapeutic strategy. The Rowe scores and Constant-Murley scores were used to evaluate functional outcomes in our study. The results showed consistent improvement in both scores across all treatment groups and follow-up time points. Notably, the arthroscopic repair and BT groups consistently demonstrated higher scores compared to the conservative treatment group, indicating better functional outcomes. However, the differences between the groups were not statistically significant. These findings suggest that, while conservative treatment may be a viable option for some patients, surgical interventions such as arthroscopic repair and BT can lead to improved functional outcomes. The decision to proceed with surgery should consider the individual patient's needs, preferences, and the severity of the SLAP lesion [13-15].

Furthermore, the results of our study align with previous research examining treatment options for SLAP lesions. Conservative treatment, including rest, physical therapy, and non-steroidal anti-inflammatory drugs (NSAIDs), has been shown to provide pain relief and functional improvement in some patients, However, surgical interventions have gained popularity due to their potential to address the underlying structural abnormalities and provide more significant and long-lasting benefits [16].

Arthroscopic labral repair is a commonly performed procedure for SLAP lesions and has shown promising outcomes in terms of pain relief and functional improvement. Our study corroborates these findings, demonstrating a progressive increase in pain relief and functional improvement over time in the repair group. The repair group exhibited consistently higher Rowe scores and Constant-Murley scores compared to the conservative treatment group, indicating better shoulder function. These results support the effectiveness of arthroscopic repair in addressing SLAP lesions and restoring shoulder stability [10,17]. BT, another surgical option, involves the detachment and reattachment of the long head of the biceps tendon to an alternative location. This procedure aims to alleviate symptoms associated with biceps pathology and SLAP lesions [17]. Our study found that patients who underwent BT experienced substantial pain relief and functional improvement. The tenodesis group consistently exhibited the highest pain relief and functional improvement scores across all follow-up time points, indicating superior outcomes compared to the conservative treatment and repair groups. These findings suggest that BT may be a valuable treatment option for patients with SLAP lesions, especially those with concomitant biceps pathology [17-19].

Arthroscopic labral repair is a commonly performed procedure for SLAP lesions and has shown promising outcomes in terms of pain relief and functional improvement. BT, another surgical option, involves the detachment and reattachment of the long head of the biceps tendon to an alternative location. This procedure aims to alleviate symptoms associated with biceps pathology and SLAP lesions [11]. Our study found that patients who underwent BT experienced substantial pain relief and functional improvement. The BT group consistently exhibited the highest pain relief and functional improvement scores across all follow-up time points, indicating superior outcomes compared to the conservative treatment and repair groups. These findings suggest that BT may be a valuable treatment option for patients with SLAP lesions, especially those with concomitant biceps pathology [11,12].

In more recent articles, the benefits and outcomes of these treatment options have been further elucidated. A study by Parnes et al. highlights that, while arthroscopic labral repair provides substantial relief in the short term, some patients might experience recurrent symptoms after a few years, emphasizing the importance of long-term follow-up [20]. On the other hand, a prospective study by Zhu et al. [21] affirms the superior results of BT, especially in athletes and those with higher demands on their shoulders. The study further notes that BT offers a more definitive solution for SLAP lesions with associated biceps pathology, resulting in fewer post-operative complications and revisions compared to labral repair alone.

Comparatively, our results bolster the findings of these newer articles, showcasing consistent improvements in the BT group across all measured outcomes. Nevertheless, it is essential to note that individualized treatment decisions should be made, taking into account the patient's specific needs, the severity of the lesion, and associated pathologies. Furthermore, a review by Thayaparan et al. [22] underscores the importance of a rigorous rehabilitation protocol post-surgery, regardless of the chosen intervention. They argued that the success of both labral repair and BT is not merely surgical; post-operative care plays a pivotal role in determining the final outcomes.

This highlights the need for doctors to focus not only on the surgery but also on the post-operative recovery strategy. It is critical to understand that the dynamic field of SLAP lesion treatments is continually evolving. Constant review and comparison with newer studies will ensure that patients receive the best possible care based on the most recent evidence [20-23]. The strengths of our study include the prospective follow-up of patients for at least two years, which allowed for a comprehensive evaluation of the treatment outcomes. We used validated outcome measures, such as VAS scores, Rowe scores, and Constant-Murley scores, to ensure objective and reliable assessments. Additionally, our study included a homogeneous patient population meeting strict inclusion and exclusion criteria, minimizing potential confounding factors [7,24].

It is important to note that the study had some limitations. First, the sample size was relatively small, with a total of 60 patients divided into three treatment groups. A larger sample size would provide more statistical power and enhance the generalizability of the findings, the study had a relatively short follow-up period of two years. Long-term outcomes beyond two years should be considered to assess the durability of the treatment effects. Furthermore, the absence of a control group receiving no treatment, or a placebo limits the ability to directly compare the treatment groups. Future studies should address these limitations by employing larger sample sizes, prospective designs, and randomization to enhance the validity of the results.

## Conclusions

Our paper shows the importance of tailoring treatments to individual patients and emphasizes the importance of a personalized medical approach, echoing the global shift towards more individual-centric healthcare. The surgical strategy seems to lead to a better outcome and better pain control in long-term follow-up. Our study not only provides conclusive insights but also illuminates areas for future investigation, underscoring the need for a deeper delve into the long-term efficacy of conservative treatments and the potential advantages of combining treatments.
